# 826. Decision to Treat *Pneumocystis Jirovecii* Pneumonia by Combining Molecular Diagnostics and Patient Specific Factors

**DOI:** 10.1093/ofid/ofad500.871

**Published:** 2023-11-27

**Authors:** Anna W Maro, Jennifer Cuellar-Rodriguez, Ahnika Kline, Sanchita Das, Sally Hunsberger

**Affiliations:** National Institute of Allergy and Infectious Diseases, National Institutes of Health, Bethesda, Maryland; National Institute of Allergy and Infectious Diseases, National Institutes of Health, Bethesda, Maryland; NIH, Bethesda, Maryland; National Institutes of Health, Bethesda, Maryland; National Institute of Allergy and Infectious Diseases, Bethesda, Maryland

## Abstract

**Background:**

*Pneumocystis jirovecii* pneumonia (PJP) diagnosis on immunocompromised patients with or without HIV has changed over time. Molecular methods have substituted microscopic methods that can be subjective, labor intensive, and require expertise. Additionally, they can be falsely negative in patients with lower fungal burden, such as transplant recipients and other immunocompromised hosts. Due to optimization of resources, our laboratory has now opted to offer only PCR based testing. We reviewed the clinical and laboratory data at our center, for PJP diagnosis prior to this change, given that we have concurrent results from a direct fluorescent antibody (DFA) assay and PCR test on samples for 16 years.

**Methods:**

We utilized data from retrospective chart review of 402 patients with a positive PJP PCR and a simultaneous DFA assay. Complete medical records were available for 302 patients. We included baseline characteristics by age, sex, race/ethnicity, and comorbid condition such as hematological malignancy diagnosis of *PJP*, solid malignancy, HIV, primary immunodeficiencies, and other chronic illnesses. Samples were collected by BAL or induced sputum. Concordance of the two tests was recorded; proportions of underlying comorbidities, use of prophylaxis and having received treatment was also recorded in relation to the concordance of both tests. Mortality within 30 and 90 days of sample collection was compared with corresponding Ct values, and DFA factors.

**Results:**

302 patients were included in the analysis (see table 1); 72% had a discordant result. 69% of patients with a discordant result were treated, compared to 99% of those with a concordant result. Patients with hematological malignancy were more represented and were more likely to have received treatment when discordant, than PLWH or other groups. The 30 and 90-day mortality was numerically similar among both groups.

**Table 1.**
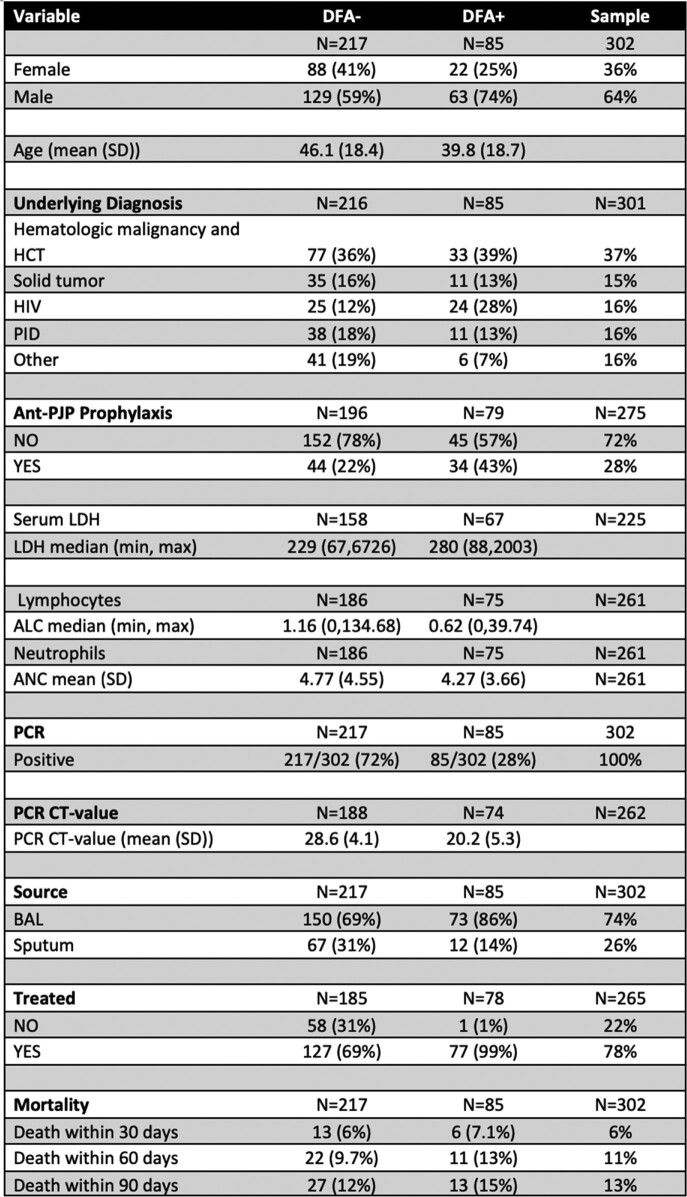
Clinical and laboratory characteristics of patients with a positive PJP PCR and a simultaneous DFA assay. Variables are expressed as proportions, unless otherwise specified.

**Conclusion:**

PCR is a very sensitive tool to detect PJP, and in the era of decreased availability microscopy studies, clinical data evaluation such as patient population, and net state of immunosuppression, in addition to a positive test, will need to be considered when deciding who requires treatment.

**Disclosures:**

**All Authors**: No reported disclosures

